# GPla Polymorphisms Are Associated with Outcomes in Patients at High Cardiovascular Risk

**DOI:** 10.3389/fcvm.2017.00052

**Published:** 2017-08-21

**Authors:** Dominik Rath, Elke Schaeffeler, Stefan Winter, Semjon Levertov, Karin Müller, Michal Droppa, Fabian Stimpfle, Harald F. Langer, Meinrad Gawaz, Matthias Schwab, Tobias Geisler

**Affiliations:** ^1^Department of Cardiology, University Hospital Tuebingen, Tuebingen, Germany; ^2^Dr. Margarete-Fischer-Bosch Institute of Clinical Pharmacology, Stuttgart, Germany; ^3^University of Tuebingen, Tuebingen, Germany; ^4^Department of Clinical Pharmacology, University Hospital Tuebingen, Tuebingen, Germany; ^5^Department of Pharmacy and Biochemistry, University of Tuebingen, Tuebingen, Germany

**Keywords:** integrin alpha2, polymorphism, single nucleotide, thromboembolism, coronary artery disease, clinical study

## Abstract

**Background:**

Platelet membrane glycoprotein receptors mediate thrombus formation. GP Ia/IIa is an essential platelet integrin receptor. Single-nucleotide polymorphisms (SNPs) of the GP Ia/IIa gene alter GP Ia/IIa expression; however, their influence on cardiovascular disease remains unclear. This study aimed to investigate the effect of the GP Ia/IIa SNPs rs1126643 and rs1062535 on clinical outcomes in a large collective including high-risk patients with cardiovascular disease.

**Methods and results:**

GP Ia SNP analysis was performed in 943 patients with symptomatic coronary artery disease. All patients were tracked for all-cause death, myocardial infarction, and ischemic stroke for 360 days. Homozygous carriers of the minor allele showed significantly worse event-free survival when compared with major allele carriers in the complete collective as well as in the subset of high-risk patients (carrying all of the following three risk factors: diabetes type II, hypertension, and hyperlipidemia). There was no significant difference in the subset of low-risk patients (carrying none of the three risk factors).

**Conclusions:**

GPla SNPs are associated with cardiovascular prognosis especially in high-risk patients. Identification of GPIa SNPs is of importance to tailor therapies in patients at already high cardiovascular risk.

## Introduction

Platelet membrane glycoprotein receptors (GP) mediate thrombus formation leading to generation of platelet thrombi that are involved in the development of acute ischemic events such as acute coronary syndrome (ACS) and ischemic stroke (1, 2). Among the essential platelet integrin receptors is GP Ia/IIa (also known as integrin α2β1). GP Ia/IIa is crucial for adhesion of platelets to collagen type I even under high shear rates typical for arterial vessels (3, 4). Consequently, deficiency of GP Ia/IIa, either acquired or congenital, leads to bleeding diathesis (5). The integrin, alpha 2 gene (ITGA2), which is coding for GP Ia/IIa, is located on chromosome 5q23-31. A single-nucleotide polymorphism (SNP) of the ITAG2 gene, that alters GP Ia/IIa expression, has been identified as GPla C807T (rs1126643). Carriers of the TT genotype (homozygous carriers of minor allele) express the highest levels, CT carriers (heterozygotes) intermediate levels, and CC carriers (homozygous carriers of major allele) the lowest levels of GPIa/IIa (6, 7). A second SNP in the ITAG2 gene (rs1062535) is associated with risk of bleeding after cardiac surgery (8). One could argue that analogous to bleeding diathesis, elevated levels of GP Ia/IIa lead to a higher risk of arterial thrombosis, therefore supporting the hypothesis that carriers of the TT or CT genotype suffer from increased incidence of myocardial infarction (MI) or ischemic stroke. Several case–control studies were launched to investigate this hypothesis, which however led to controversial results. Carriers of the T allele were reported to have an increased risk of MI and ischemic stroke in some studies, whereas other studies could not show a significant difference (7, 9–13). Santoso et al. report that the T807 allele is associated with incidence of MI in younger patients (9). Analogous to this observation, there might be a significant influence of GP Ia SNPs on the development of ischemic stroke in younger patients (14). On the other hand, GP Ia rs1126643 failed to show a significant effect on the risk of restenosis after stenting in patients with stable or unstable coronary artery disease (CAD) excluding patients with MI (15). The same group showed that GP Ia rs1126643 did not have a relevant impact on major adverse events including death, MI, and urgent target vessel revascularization up to 30 days after coronary stenting. A recent meta-analysis investigating 19 studies could not find evidence supporting a positive relation between either CAD or MI and the rs1126643 polymorphism of the ITGA2 gene (16). One possible explanation could be given by the hypothesis that the GP Ia rs1126643 polymorphism does not influence development of coronary atherosclerosis as such but might lead to increased platelet thrombogenicity, therefore leading to ischemic events primarily in younger patients without significant CAD (17). Several studies indicate that GP Ia polymorphisms are associated with adverse cardiovascular events in younger patients, whereas little data suggest an association between GP Ia polymorphisms and patients at high cardiovascular risk. So far, the influence of GP Ia rs1126643 and rs1062535 on cardiovascular disease remains a matter of debate. Hence, this study aimed to investigate the influence of the GP Ia SNPs rs1126643 and rs1062535 on clinical outcomes including all-cause death, MI, and ischemic stroke in a large heterogeneous collective including high-risk patients with cardiovascular disease.

## Materials and Methods

### Subjects

GP Ia SNP analysis was performed in a previously described cohort of 943 consecutive patients with stable CAD and ACS including non-ST-elevation myocardial infarction and ST-elevation myocardial infarction (18). CAD was defined as narrowing of at least one coronary artery ≥50% (19). We defined stable CAD as a clinical syndrome characterized by discomfort in the chest, jaw, shoulder, back, or arms, typically elicited by exertion or emotional stress and relieved by rest or nitroglycerin (20). ACS was defined as worsening of angina (unstable angina pectoris) and acute MI. Arterial hypertension was defined as ≥140 mmHg in systolic and ≥90 mmHg in diastolic blood pressure (21). Hyperlipidemia was defined as baseline LDL cholesterol ≥160 mg/dl and triglycerides ≥200 mg/dl (22). Diabetes mellitus type II was defined as fasting plasma glucose ≥125 mg/dl or 2-h plasma glucose ≥200 mg/dl during standardized 75-g oral glucose tolerance test or symptoms of hyperglycemia plus non-fasting plasma glucose ≥200 mg/dl or HbA1c ≥6.5% (23). For subgroup analysis, we defined patients at very high cardiovascular risk suffering from all three cardiovascular risk factors such as diabetes mellitus type II, arterial hypertension, and hyperlipidemia, whereas a low-risk cohort was defined as patients suffering from none of the aforementioned risk factors. All subjects gave written informed consent. Patients were admitted to the Department of Cardiology of the University of Tuebingen, Germany. The study was approved by the institutional ethics committee (Ethik-Kommission an der Medizinischen Fakultät der Eberhard-Karls-Universität und am Universitätsklinikum Tübingen) (270/2011BO1) and complies with the declaration of Helsinki and the good clinical practice guidelines (24–26).

### Genotyping of GP Ia Variants

Genotyping for this GP Ia variants was performed as previously described by matrix-assisted laser desorption/ionization time-of-flight mass spectrometry (MALDI-TOF MS) using the MassARRAY^®^ Compact system (Sequenom, CA, USA) (18). Study personnel assessing outcome was blinded to the case status of the study participants during the entire genotyping process.

### Follow-up

All patients were tracked after initial PCI for clinical events including all-cause death, MI and ischemic stroke for 360 days after study inclusion. An acute MI was diagnosed by a rise and/or fall of cardiac biomarker values (cardiac troponin) with at least one value above the 99th percentile upper reference limit and with at least one of the following: symptoms of ischemia, new or presumed new significant ST-segment–T wave changes or new left bundle branch block, development of pathological Q waves in the ECG, imaging evidence of new loss of viable myocardium or new regional wall motion abnormality or identification of an intracoronary thrombus by angiography (27). CNS infarction and ischemic stroke was defined as brain, spinal cord, or retinal cell death attributable to ischemia, based on pathological, imaging, or other objective evidence of cerebral, spinal cord, or retinal focal ischemic injury in a defined vascular distribution; or clinical evidence of cerebral, spinal cord, or retinal focal ischemic injury based on symptoms persisting ≥24 h or until death, and other etiologies excluded (28). The combined primary endpoint consisted of either time to death, MI, or ischemic stroke. Secondary endpoints included the single events of all-cause death, MI, and ischemic stroke. 71 patients were lost to follow-up (7.5%). The patients lost to follow-up did not significantly differ in their baseline characteristics as compared to the group remaining in the study (*n* = 872). Moreover, both groups showed similar distributions regarding the analyzed GP Ia variants. Follow-up for the primary combined endpoint (CE) was performed until first occurrence of one of the pre-defined endpoints. Follow-up was performed by telephone interview and/or review of patients’ charts on readmission by investigators blinded to the results of laboratory testing. Mortality, MI, and ischemic stroke were ascertained by investigating hospital discharge letters and telephone interview with the patients’ physician.

### Statistical Analysis

Statistical analyses were performed using SPSS version 21.0 (SPSS Inc., Chicago, IL, USA). Chi-square tests and Student’s ANOVA were applied as appropriate to analyze baseline characteristics. Concerning the GPIA SNPs, two different genetic models were considered for these biallelic variants: recessive and additive genetic model. In the recessive model, homozygote and heterozygote carriers of the major allele are combined and compared versus homozygote carriers of the minor allele. In the additive model, homozygote carriers of the major allele, heterogyzotes, and homozygote carriers of the minor allele are numerically coded as 0, 1, and 2, respectively, assuming a (linear) allele-dose effect. The Cochran–Armitage test was performed to test for associations between endpoints and GPIa SNPs in the additive genetic model. Cox regression analysis was applied to test for the association between GPIa SNPs (additive or recessive model) and survival (CE, all-cause death, or MI), using epidemiological factors influencing cardiovascular outcome as covariables (arterial hypertension, hyperlipidemia, diabetes mellitus type II, smoking, acetylsalicylic acid, angiotensin converting enzyme inhibitors, beta blockers, statins, age, gender, left ventricular ejection fraction, and reason of admission). The time-dependent covariate method was used to check the proportional hazard assumption of the model. Survival functions were estimated by Kaplan–Meier curves. The log-rank test was applied to compare survival functions between homozygote carriers of major allele and carriers of the minor allele (i.e., recessive model). All statistical tests were two-sided and statistical significance level was defined as 5%.

## Results

GPIa variants rs1126643 and rs1062535 were highly linked in our cohort (*D*′ = 0.996, *r* = 0.994). Hence, throughout this article, results are only presented and discussed for rs1126643.

Patients’ characteristics (age, gender, cardiovascular risk factors, co-medication) of the cohort (*n* = 943), with and without stratification according to rs1126643, are provided in Table 1.

**Table 1:** Baseline characteristics of the complete cohort (*n* = 943).

Baseline characteristics	All (*n* = 943)	Homozygous carriers of major allele (*n* = 380)	Heterozygotes (*n* = 410)	Homozygous carriers of minor allele (*n* = 151)	*p*
Age (mean ± SD)	68 (±13)	68 (±12)	67 (±13)	67 (±13)	0.150
No. of males	662 (70%)	270 (71%)	282 (69%)	110 (73%)	0.599
**Cardiovascular risk factors**					
Arterial hypertension	760 (81%)	313 (82%)	323 (79%)	124 (82%)	0.337
Hyperlipidemia	522 (55%)	229 (60%)	212 (52%)	80 (53%)	0.048
Diabetes mellitus type II	298 (32%)	131 (35%)	117 (29%)	50 (33%)	0.187
Smoking	375 (40%)	154 (41%)	158 (39%)	62 (41%)	0.743
**Clinical factors**					
Left ventricular ejection fraction% (mean ± SD)	50 (±11)	50 (±11)	51 (±11)	49 (±11)	0.109
Creatinine (mg/dl) (mean ± SD)	1.0 (±0.7)	1.1 (±0.8)	1.0 (±0.5)	1.1 (±0.6)	0.156
**Medication on admission**					
Acetylsalicylic acid	495 (53%)	208 (55%)	212 (52%)	74 (49%)	0.533
Clopidogrel	108 (12%)	43 (11%)	52 (13%)	13 (9%)	0.420
Prasugrel	17 (2%)	7 (2%)	9 (2%)	1 (1%)	0.487
Ticagrelor	38 (4%)	12 (3%)	20 (5%)	6 (4%)	0.468
Oral anticoagulation	80 (9%)	30 (8%)	33 (8%)	16 (11%)	0.534
Angiotensin converting enzyme inhibitors	395 (42%)	163 (43%)	170 (42%)	60 (40%)	0.825
Angiotensin II receptor antagonists	173 (18%)	82 (22%)	70 (17%)	21 (14%)	0.085
Ca-channel inhibitors	180 (19%)	72 (19%)	77 (19%)	31 (21%)	0.874
Beta blockers	528 (56%)	213 (56%)	226 (55%)	87 (58%)	0.834
Statins	425 (45%)	178 (47%)	176 (43%)	70 (46%)	0.509
**Reason of admission**					
Acute coronary syndrome (ACS)	388 (41%)	161 (42%)	157 (38%)	70 (46%)	0.508
Stable coronary artery disease (CAD)	373 (40%)	144 (38%)	172 (42%)	55 (36%)	
Other^a^	181 (19%)	75 (20%)	81 (20%)	26 (17%)	
Missing values for rs1126643	2 (0.2%)				

*^a^Consists of myocarditis, non-ischemic cardiomyopathy, valve stenosis or insufficiency, heart rhythm events, suspected CAD, and pre-surgery*.

Number and categories of events are shown in Table 2.

**Table 2:** Events and incident rate (IR)/100 person years (PY) in the overall cohort (*n* = 870).

Variable	No. of events (rs1126643: hc of major alleles/heterozygotes/hc of minor allele)	IR/100 PY (rs1126643: hc of major alleles/heterozygotes/hc of minor allele)	*p*
Combined endpoint	98 (43/32/23)	11.3 (11.0/7.8/15.2)	0.500
All-cause death	51 (20/18/13)	5.9 (5.3/4.4/8.6)	0.250
Myocardial infraction	54 (25/15/14)	6.2 (6.6/3.7/9.3)	0.630
Ischemic stroke	13 (6/6/1)	1.5 (1.6/1.5/0.7)	0.500

*hc, homozygous carrier*.

All patients were followed up after initial PCI. By using an additive genetic model, we could not find significant associations between rs1126643 and the CE or the secondary endpoints in the overall cohort (Table 2). Rs1126643 was significantly correlated with the CE in patients who suffered from diabetes type II, arterial hypertension, and hyperlipidemia (defined as high-risk cohort), but not in patients without any of these risk factors (defined as low-risk cohort) (see Table 3). However, power of these analyses was low due to limited number of cases (*n* = 163 and *n* = 83, respectively) and low event rate.

**Table 3:** Events and incident rate (IR)/100 person years (PY) in the high- and low-risk cohort.

Variable	No. of events (high risk) *n* = 163 (hc of major alleles/heterozygotes/hc of minor allele)	IR/100 PY (high risk) (hc of major alleles/heterozygotes/hc of minor allele)	*p* (high risk)
Combined endpoint	19 (7/4/8)	11.7 (8.6/7.7/26.7)	0.027

**Variable**	**No. of events (low risk) *n* = 83 (hc of major alleles/heterozygotes/hc of minor allele)**	**IR/100 PY (low risk) (hc of major alleles/heterozygotes/hc of minor allele)**	***p* (low risk)**

Combined endpoint	8 (3/3/2)	9.8 (9.7/7.9/15.4)	0.689

*hc, homozygous carrier*.

Multivariate Cox regression analysis revealed that rs1126643 was not significantly associated with the CE as well as MI after adjustment for epidemiological factors, using an additive genetic model (Table 4). However, in the recessive genetic model (i.e., comparing carriers of major allele versus homozygous carrier of minor allele), we could find significant independent associations between the GPIa C807T variants and both, the CE (hazard ratio 1.95, *p* = 0.011) and MI (hazard ratio 2.11, *p* = 0.028) (Table 5). As shown in Table 6, we could find significant independent associations between the GPIa C807T variants and the CE (hazard ratio 3.29, *p* = 0.024) (Table 6).

**Table 4:** Cox regression analyses for the combined endpoint (CE) or myocardial infarction (MI) as dependent variable, rs1126643 (additive genetic model) as independent variable, and clinical factors as covariates in the overall cohort of cardiovascular patients (*n* = 870).

Cardiovascular risk factors	Hazard ratio (CE) (95% CI)	Hazard ratio (MI) (95% CI)	*p* (CE)	*p* (MI)
Arterial hypertension (yes/no)	1.02 (0.52–2.00)	1.50 (0.56–4.03)	0.963	0.419
Hyperlipidemia (yes/no)	0.70 (0.44–1.12)	0.77 (0.41–1.44)	0.135	0.412
Diabetes mellitus type II (yes/no)	1.22 (0.76–1.95)	0.89 (0.47–1.66)	0.404	0.704
Smoking (yes/no)	0.72 (0.42–1.23)	0.56 (0.28–1.12)	0.232	0.102
**Medication on admission**				
Acetylsalicylic acid (yes/no)	2.15 (1.25–3.72)	2.37 (1.13–4.98)	0.006	0.023
Angiotensin converting enzyme inhibitors (yes/no)	0.76 (0.47–1.23)	0.69 (0.37–1.29)	0.266	0.242
Beta blockers (yes/no)	1.28 (0.74–2.21)	1.81 (0.86–3.82)	0.377	0.121
Statins (yes/no)	0.78 (0.47–1.31)	0.93 (0.47–1.84)	0.347	0.837
**Clinical factors**				
Age	1.05 (1.02–1.08)	1.04 (1.01–1.08)	<0.001	0.18
Gender (female/male)	0.96 (0.58–1.59)	0.60 (0.29–1.25)	0.877	0.173
Left ventricular ejection fraction %	0.96 (0.94–0.98)	0.96 (0.94–0.99)	<0.001	0.003
Reason of admission (acute coronary syndrome, stable coronary artery disease, other)	1.61 (1.16–2.22)	2.30 (1.43–3.69)	0.004	0.001
GPla rs1126643 (additive genetic model)	1.16 (0.91–1.50)	1.37 (0.99–1.89)	0.230	0.059

**Table 5:** Cox regression analysis for the combined endpoint (CE) and myocardial infarction (MI) as dependent variable, rs1126643 (recessive genetic model) as independent variable, and clinical factors as covariates in the overall cohort of cardiovascular patients (*n* = 870).

Cardiovascular risk factors	Hazard ratio (CE) (95% CI)	Hazard ratio (MI) (95% CI)	*p* (CE)	*p* (MI)
Arterial hypertension (yes/no)	0.96 (0.49–1.89)	1.46 (0.54–3.94)	0.902	0.542
Hyperlipidemia (yes/no)	0.71 (0.44–1.12)	0.80 (0.43–1.48)	0.140	0.801
Diabetes mellitus type II (yes/no)	1.20 (0.75–1.92)	0.86 (0.46–1.63)	0.456	0.643
Smoking (yes/no)	0.75 (0.44–1.27)	0.57 (0.28–1.16)	0.283	0.122
**Medication on admission**				
Acetylsalicylic acid (yes/no)	2.23 (1.28–3.90)	2.48 (1.16–5.31)	0.005	0.020
Angiotensin converting enzyme inhibitors (yes/no)	0.76 (0.47–1.24)	0.68 (0.36–1.28)	0.272	0.229
Beta blockers (yes/no)	1.24 (0.71–2.15)	1.73 (0.81–3.71)	0.449	0.156
Statins (yes/no)	0.80 (0.48–1.34)	0.95 (0.48–1.87)	0.396	0.880
**Clinical factors**				
Age	1.05 (1.03–1.08)	1.04 (1.01–1.08)	<0.001	0.013
Gender (female/male)	0.97 (0.58–1.61)	0.60 (0.29–1.25)	0.894	0.173
Left ventricular ejection fraction %	0.96 (0.94–0.98)	0.96 (0.93–0.99)	<0.001	0.002
Reason of admission (acute coronary syndrome, stable coronary artery disease, other)	1.61 (1.17–2.23)	2.11 (1.46–3.82)	0.004	< 0.001
GPla rs1126643 (recessive genetic model)	1.95 (1.17–3.25)	2.11 (1.08–4.10)	0.011	0.028

**Table 6:** Cox regression analysis for the combined endpoint (CE) as dependent variable, rs1126643 (recessive genetic model) as independent variable, and clinical factors as covariates in the high-risk cohort (*n* = 163).

Medication on admission	Hazard ratio (CE) (95% CI)	*p* (CE)
Angiotensin converting enzyme inhibitors (yes/no)	0.74 (0.27–2.02)	0.559
Beta blockers (yes/no)	2.85 (0.58–13.89)	0.196
Statins (yes/no)	1.14 (0.36–3.65)	0.822
**Clinical factors**		
Age	1.06 (1.00–1.13)	0.054
Gender (female/male)	1.51 (0.50–4.52)	0.463
Left ventricular ejection fraction%	0.97 (0.93–1.02)	0.262
Reason of admission (acute coronary syndrome, stable coronary artery disease, other)	1.61 (0.71–3.67)	0.255
GPla rs1126643 (recessive genetic model)	3.78 (1.39–10.28)	0.009

**Figure 1:**
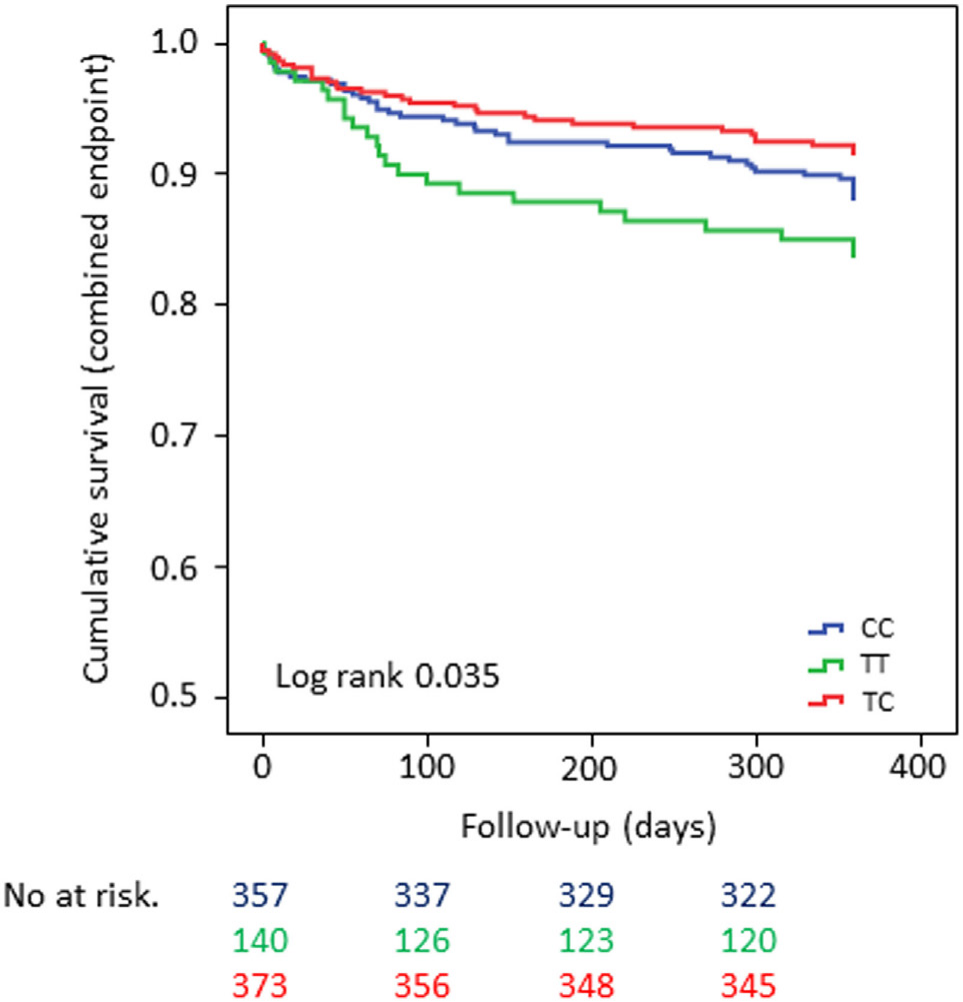
Kaplan–Meier curves showing cumulative survival (combined endpoint all-cause death and/or Ml and/or ischemic stroke) stratified according to GP la rs1126643 homozygous carriers of major allele, heterozygous and homozygous carriers of minor allele. No. at risk: blue = homozygous carriers of major allele, green = homozygous carriers of minor allele, red = heterozygous carriers of minor allele.

**Figure 2:**
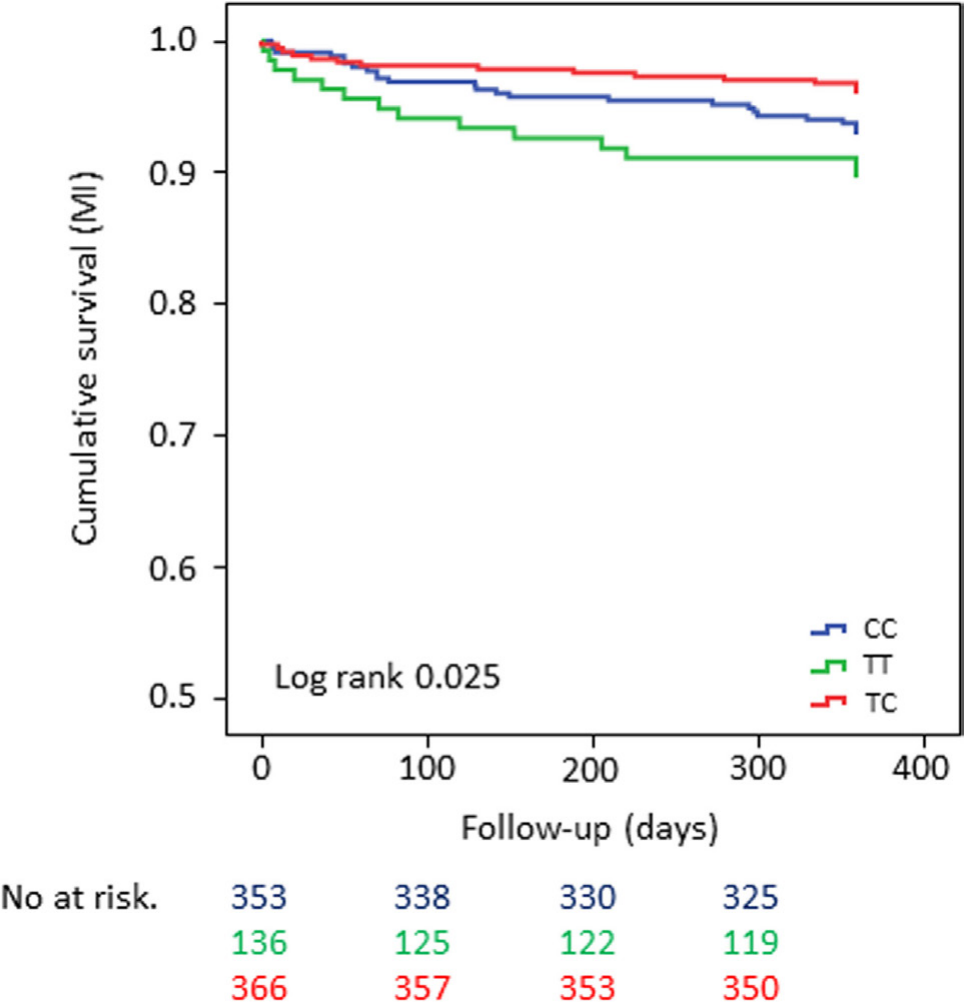
Kaplan–Meier curves showing cumulative survival [myocardial infarction (MI)] stratified according to GP la rs1126643 homozygous carriers of major allele, heterozygous and homozygous carriers of minor allele. No. at risk: blue = homozygous carriers of major allele, green = homozygous carriers of minor allele, red = heterozygous carriers of minor allele.

**Figure 3:**
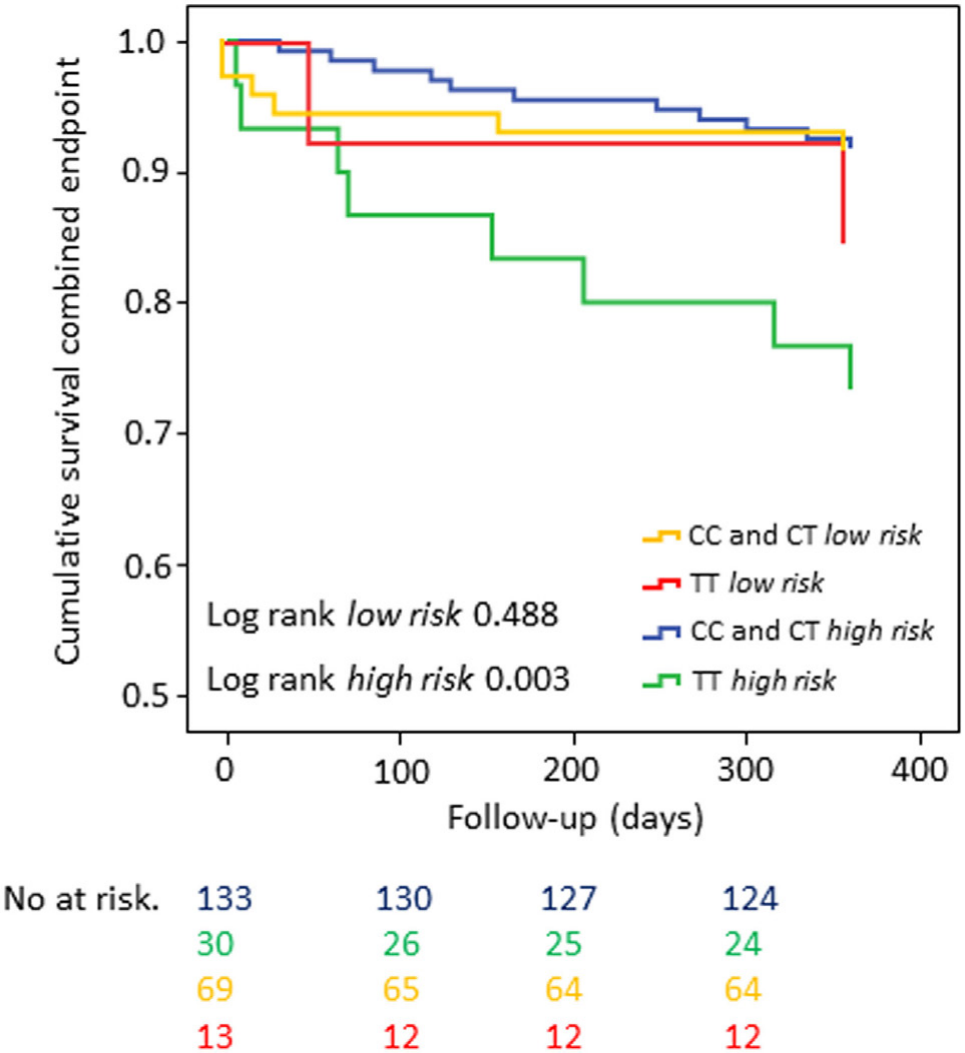
Kaplan–Meier curves showing cumulative survival (combined endpoint) stratified according to GP la rs1126643 homozygous and heterozygous carriers of major allele versus homozygous carriers of minor allele in a patient collective at high cardiovascular risk. No. at risk: blue = homozygous and heterozygous carriers of major allele (*high risk*), green = homozygous carriers of minor allele (*high risk*), yellow = homozygous-and heterozygous carriers of major allele (*low risk*), red = homozygous carriers of minor allele (*low risk*).

In the overall cohort, patients who were homozygous carriers of the minor allele showed significantly shortened time to event for the CE when compared with carriers of the major allele (univariate analysis: log-rank test *p*-value = 0.035 for CE and log-rank test *p*-value = 0.025 for MI, respectively) (Figures 1 and 2). The same held true in the high-risk cohort (log-rank test *p*-value = 0.003 for CE), whereas rs1126643 was not significant associated with the CE in the low-risk cohort (log-rank test *p*-value = 0.448 for CE) (Figure 3).

## Discussion

The major finding of the present study is that GPla rs1126643 and rs1062535 polymorphisms are associated with outcomes in patients with cardiovascular disease in particular in high-risk patients (e.g., in patients with diabetes type II, arterial hypertension, and hyperlipidemia).

Glycoprotein Ia is a component of the GP Ia/IIa complex, a collagen receptor on platelets. This complex is a member of the integrin family and contributes to primary hemostasis after vessel injury (1, 2). The SNP GPIa C807T might alter expression levels of the GP Ia/IIa receptor on the platelet surface, therefore possibly influencing hazard of the patient for thromboischaemic events (6, 7). The hypothesis that GPIa C807T polymorphisms might affect thromboembolic complications in patients with cardiovascular disease led to a number of studies. However, available data provide conflicting results. A meta-analysis by Tsantes et al. could not find evidence supporting an association of both, CAD and MI with the C807T polymorphism (16). In this meta-analysis, studies were included that investigated the impact of the polymorphism on a cross-sectional basis comparing patients with first onset of CAD and/or MI with a control group in a general population, or subjects in a hospital or a clinic showing no manifestation or having no history of CAD. In our study, we did not aim to investigate associations of GP Ia C807T polymorphisms with primary onset of CAD since most of our patients already suffered from CAD at study inclusion. We could show that GP Ia polymorphisms are significantly associated with recurrent events, i.e., a composite endpoint of all-cause death, MI, and/or ischemic stroke as well as the single endpoint MI. Interestingly, as shown with *post hoc* tests, the incidence rates did not correlate with the number of T-alleles as suggested by increasing GP Ia/IIa receptor density depending on the number of T-alleles. We could not find a significant increase in endpoints in dependence of the number of T-alleles. Thus, our results suggest that an increase of GPIa/IIa receptor density does not necessarily result in an increase of thrombo ischemic events even though homozygous carriers of the minor allele showed an increase in events when compared to both, homozygous carriers of the major allele and heterozygous carriers of the minor allele. A possible explanation for these unexpected findings might be a study collective that differs in their risk for developing thromboembolic events by differential manifestations of thromboinflammation. However, after adjusting for risk factors, the GP Ia polymorphisms remained independently associated with the CE as well as MI. Of note, we found the strongest association in patients with a very high cardiovascular risk profile. It is tempting to speculate that the prognostic role of the GP Ia polymorphisms increases with the baseline cardiovascular risk of the patients. Recently, it was discovered that GPIa rs1126643 T-allele carriage increases risk of MACCE after coronary artery bypass surgery. The patient collective in this study suffered to a large extent of 3-vessel disease (88%) and several cardiovascular risk factors (hypertension 67%, hyperlipidemia 68%, and diabetes mellitus 33%), which predestines them for future cardiovascular events. Patients with hypertension showed a significantly increased rate of major adverse cardiovascular events when compared to patients without hypertension (29). Heeschen et al. have previously shown that elevated levels of soluble CD40 ligand (sCD40L), which is released after platelet activation, are associated with increased risk of cardiovascular events in patients with CAD (30). This is interesting because the T-allele of rs1126643 has been demonstrated to be an independent predictor for the release of sCD40L during the acute phase of MI, an effect that persisted in the same patients 1 year after the event. The same study group showed that the presence of the rs1126643 T-allele was associated with increased levels of sCD40L in healthy subjects, but only in the presence of high vWF levels (31). However, these results could not be confirmed in a subsequent study of a different group (32). Furthermore, GP Ia polymorphisms could therefore be used to identify patients that would benefit from a more stringent secondary prophylaxis, e.g., prolonging the duration of dual antiplatelet therapy beyond 12 months after stent implantation in ACS patients. It has been previously demonstrated that risk of ischemic stroke is associated with the expression of the T807 allele (14). In our study, we could not show a difference in the incidence of ischemic stroke according to GP Ia C807T SNPs, probably explained by the small number of events. Santoso et al. found an association between MI and the presence of the T807 allele. However, this association was only significant when the authors investigated patients below the age of 62 years (9). In our study, we can demonstrate an association between MI and GP Ia SNPs in a heterogeneous patient collective with cardiovascular disease. Furthermore, homozygous carriers of the minor allele showed the highest event rate of MI.

To summarize, our study investigated an association of the GP Ia polymorphisms with outcomes in patients with cardiovascular disease. We could find a significant association of the GP Ia polymorphisms with the CE and the single endpoint MI. We found the strongest associations in patients at very high cardiovascular risk. These findings remained significant after adjustment for risk factors.

Thus, the role of the GP Ia/IIa polymorphisms for the prognosis of cardiovascular disease remains a matter of debate, but might help to individualize risk-based strategies for secondary prophylaxis especially in high-risk patients.

### Limitations

We are aware that our results are only hypothesis generating. Our study has certain limitations mainly due to its observational character, the moderate sample size, low event rate, and lost to follow-up. Especially the subgroup analysis of the high-risk cohort has low power due to low sample size. Furthermore, we are fully aware that the literature research does not replace a prospective validation study. However, we believe that the publications mentioned in the discussion section are highly confirmative for an association of distinct GPIa genetic variants and cardiovascular events in different high-risk populations. We did not account for other potential confounders including biomarkers that have previously been associated with outcome in cardiovascular cohorts. Since the study cohort consisted of mostly Caucasian individuals, it might be difficult to generalize the results for other ethnicities.

## Ethics Statement

All subjects gave written informed consent. Patients were admitted to the Department of Cardiology of the University of Tuebingen, Germany. The study was approved by the institutional ethics committee (Ethik-Kommission an der Medizinischen Fakultät der Eberhard-Karls-Universität und am Universitätsklinikum Tübingen) (270/2011BO1) and complies with the declaration of Helsinki and the good clinical practice guidelines.

## Author Contributions

DR: patient selection, statistical analysis, and drafting of the manuscript. ES: genotyping and drafting of the manuscript. SW: statistical analysis and drafting of the manuscript. SL: patient baseline characteristics and follow-up. HL: Critical revision. KM: critical revision. MD: critical revision. FS: critical revision. MG: critical revision and funding. MS: critical revision and funding. TG: drafting of the manuscript and funding.

## Conflict of Interest Statement

The authors declare that the research was conducted in the absence of any commercial or financial relationships that could be construed as a potential conflict of interest.
